# West Texas Medical Association

**Published:** 1890-12

**Authors:** James Kennedy

**Affiliations:** Secretary


### Society Notes.

### WEST TEXAS MEDICAL ASSOCIATION.

*Editor Daniel's Texas Medical Journal:*

The regular quarterly meeting of the West Texas Medical Association convened in the city of San Antonio October 30th inst., at 10 a. m. Dr. G. W. Kerr, President, in the chair. The morning session was occupied with routine business and receiving applications for membership.

The afternoon session was spent in a most edifying manner. Several meritorious contributions to medical science were read and ably discussed by members present. The essays presented were without exception of high character, and reflected credit upon their respective authors.

Dr. A. S. McDaniel read a paper* on Appendicitis, discussing the pathology, course and treatment of this disease. Dr. R. Menger reported a case of aneurysm of the palmar arch in which after twice ligating the radial artery amputation was necessitated on account of gangrene.

Dr. H. J. Trolinger read a paper on hay-fever, the chief feature of which was a consideration of its ætiology. He does not accept the neurotic origin—does not believe the theory that it is a "neurosis" a tenable one.

The President, Dr. G. W. Kerr, read a paper on per oxide of hydrogen, extolling the anti-suppuratiue virtues of this potent but unstable compound.

It goes without saying that the discussion following the read-

*Published in this issue.

ing of these papers was both instructive and entertaining. The evening session was even better attended than either the morning or afternoon. As soon as the meeting was called to order a resolution† was introduced for the unanimous endorsement of Dr. R. M. Swearingen for state health officer. The applause which this resolution received left no doubt as to the unanimity on that score.

The election of officers came next in order, and the following gentlemen were duly elected by acclamation:

Dr. J. V. Spring, San Antonio, President; Dr. A. Garwood, New Braunfels, First Vice-President; Dr. F. Terrell, San Antonio, Second Vice-President; Dr. James Kennedy, San Antonio, Secretary and Treasurer.

After hearing the report of the faithful and zealous retiring Secretary, Dr. Berrey, the question as to the place of next meeting was voted on and it was decided to meet again in the hospitable city of the Alamo. The meeting then adjourned but was shortly thereafter re-convened in the banquet hall where a delightful repast awaited us—and in so much as we have been told that "man should not live by bread alone" other palatable accessories were in waiting. We ate, drank and made merry, then dispersed to our respective abodes.

The next quarterly meeting will be held in this city (San Antonio) Monday, February 1, 1891.

JAMES KENNEDY, M. D., Secretary.

---

### CHEROKEE COUNTY MEDICAL SOCIETY.

*Editor Daniel's Texas Medical Journal:*

The Cherokee County Medical Society met in regular session at Jacksonville last month. Owing to the ill health of Dr. McCord the committee on collective investigation made no report. Three regular papers, however, were read. One by Dr. Evans,

†Published in the November number of the Journal.—ED.

